# *Periplaneta americana* Extracts Accelerate Liver Regeneration *via* a Complex Network of Pathways

**DOI:** 10.3389/fphar.2020.01174

**Published:** 2020-07-31

**Authors:** Yingying Zou, Meiyan Zhang, Di Zeng, Yonghua Ruan, Lijuan Shen, Zhihao Mu, Jiangmeng Zou, Chenjian Xie, Zhihong Yang, Zhongyi Qian, Ruobing Xu, Shude Li, Qiang Kang, Hao Zou, Songling Zhao, Lixin Liu, Kun Wang, Xie Wang, Xiaowen Zhang

**Affiliations:** ^1^Department of Pathology and Pathophysiology, Kunming Medical University, Kunming, China; ^2^Department of Hepatobiliary Surgery, Second Affiliated Hospital of Kunming Medical University, Kunming, China; ^3^Department of Morphological Laboratory, Kunming Medical University, Kunming, China; ^4^Department of Biochemistry and Molecular Biology, Kunming Medical University, Kunming, China

**Keywords:** bioinformatics, liver regeneration, *Periplaneta americana* extracts, proliferation, signaling pathways

## Abstract

Successful recovery from hepatectomy is partially contingent upon the rate of residual liver regeneration. The traditional Chinese medicines known as *Periplaneta americana* extracts (PAEs) positively influence wound healing by promoting tissue repair. However, the effect of PAEs on liver regeneration is unknown. We used a mouse liver regeneration model after 70% partial hepatectomy (PH) and a hepatocyte culture to determine whether PAEs can promote liver regeneration as effectively as skin regeneration and establish their modes of action. L02 cells were divided into serum-starved control (NC) and three PAEs (serum starvation + 0.1 mg/ml, 0.5 mg/ml, or 1 mg/ml PAEs) groups. L02 cell proliferation was assessed at 24 h, 48 h, and 72 h by CCK-8 assay. Forty male C57 mice were randomly divided into control (NC), normal saline (NS), PAEs400 (400 mg/kg/d), and PAEs800 (800 mg/kg/d) groups (n = 10 per group). The NS and both PAEs groups were administered normal saline and PAEs, respectively, by gavage for 10 days. Two hours after the tenth gavage, the NS and both PAEs groups were subjected to 70% PH and the residual liver was harvested after 48 h. The hepatic regeneration rate was evaluated and hepatocyte proliferation was estimated by immunohistochemical (IHC) staining for Ki-67. Twelve DEG libraries (three samples per group) were prepared and sequencing was performed in an Illumina HiSeq 2000 (Mus_musculus) at the Beijing Genomics Institute. The genes expressed in the liver tissues and their expression profiles were analyzed by bioinformatics. KEGG was used to annotate, enrich, and analyze the pathways. PAEs promoted hepatocyte proliferation *in vitro* and *in vivo* and accelerated mouse liver regeneration after 70% PH. The screening criteria were fold change (FC) ≥ 2 and q-value < 0.001. We identified 1,092 known DEGs in PAEs400 and PAEs800. Of these, 153 were categorized in cellular processes. The KEGG analysis revealed that the aforementioned DEGs participated in several signaling pathways closely associated with cell proliferation including PI3K-Akt, MAPK, Apelin, Wnt, FoxO, mTOR, Ras, VEGF, ErbB, Hippo, and AMPK. It was concluded that PAEs can effectively improve liver regeneration *via* the synergistic activation of different signaling pathways.

## Introduction

The liver performs and regulates numerous physiological functions and has the ability to regenerate. Parenchymal cells or hepatocytes constitute ~80% of the liver tissue and execute most of the physiological functions of this organ. The balance of the liver consists of non-parenchymal endothelial, stellate, and Kupffer cells as well as lymphocytes (Taub, 2004). Successful recovery after partial hepatectomy (PH) depends mainly on rapid residual liver regeneration and liver function recovery. However, few clinically available drugs significantly enhance hepatocyte proliferation or liver regeneration. Therefore, the quest for new drugs or therapeutic targets that can improve liver regeneration is of great theoretical and practical importance.

Several animal models have been designed to evaluate liver regeneration. In 1931, Higgins and Anderson proposed a rat model of 2/3 hepatectomy (Nevzorova et al., 2015). It continues to be popular to this day as it involves the excision of intact liver lobes and does not damage the residual liver. In contrast, other models using toxicants such as carbon tetrachloride to induce injury to the residual liver (Mao et al., 2014). Though the excised lobes never regenerate, the residual liver tissue restores the original liver mass within ~1 week after surgery (Taub, 2004). The PH liver regeneration model is used to synchronize and evaluate cell cycle events and signal transduction *in vivo* (Fausto et al., 2006). Certain *in vitro* methods also synchronize mammalian cell cultures and are used to study regulatory mechanisms of cell cycle progression and cell proliferation. These include serum starvation/deprivation, chemically-induced cell cycle arrest, mitotic shake-off, counterflow centrifugal elutriation, and newer live cell methods. Each of these methods has inherent disadvantages. Nevertheless, serum starvation is simple, reversible, reliable, and generally applicable to mammalian cells (Langan et al., 2017) including hepatocytes.

Liver regeneration is extremely complex and involves multiple factors and pathways. Parenchymal and non-parenchymal liver cells proliferate to replace lost hepatic tissue. However, hepatocytes proliferate first (Michalopoulos and DeFrances, 2005). Liver regeneration approximately entails the priming phase (early period after PH; quiescent hepatocytes transition from G0 to G1), the proliferation phase (progression phase; DNA synthesis and hepatocyte proliferation), and the termination phase (hepatocyte proliferation stops as soon as the liver mass is restored) (Fausto, 2000; Tao et al., 2017). The earliest signals initiating the regenerative response remain to be elucidated. However, it is known that IL-6 (interleukin-6) and TNF-α (tumor necrosis factor-α) participate in the events that transform hepatocytes from G0 to G1 in the priming phase and this process is mediated by the NF-κB, JAk/STAT, and MAPK signaling pathways (Taub, 2004; Michalopoulos and DeFrances, 2005; Mao et al., 2014; Tao et al., 2017). Complete mitogens and auxiliary mitogens are involved in the proliferation phase. The former activates secondary or delayed gene responses, stimulate DNA replication, and induce hepatocyte proliferation *via* the JAk/STAT, PI3K/AKT, mTOR, ERK, and MAPK signaling pathways. Complete mitogens include TGF-α (transforming growth factor-α), HGF (hepatocyte growth factor), EGF (epidermal growth factor), and EGFR (epidermal growth factor receptor) (Taub, 2004). The latter may partially contribute to regeneration by accelerating or magnifying the effects of complete mitogens even though they are not mitogenic in hepatocytes. Auxiliary mitogens include VEGF (vascular endothelial growth factor), IGF (insulin-like growth factor), Bas (bile acids), NE (norepinephrine), and estrogen (Tao et al., 2017). The mechanisms of the termination phase are poorly understood. TGF-β1 (transforming growth factor-β1) is the best known antiproliferative factor (Tao et al., 2017). Despite extensive investigation, the exact mechanisms of liver regeneration have not yet been clarified.

Certain animal products have been used in traditional Chinese medicine (TCM). The ancient Chinese science of disease treatment has been advocated for thousands of years. *Periplaneta americana* (PA), the American cockroach, has a long history of application for the treatment of various injuries (Alves and Alves, 2011; Zhu et al., 2018). Over the past few years, physiological and pharmacological studies have demonstrated that TCM powder and extracts of *Periplaneta americana* (PAEs) (Zeng et al., 2019) have tissue repair (Yang et al., 2015; Li et al., 2016; Zhu et al., 2018), antitumor (Luo et al., 2014; Zhao et al., 2017), antibacterial (Basseri et al., 2016; Ali et al., 2017), antiviral (Li and Hu, 2003), antifungal (Yun et al., 2017), antifibrotic (Li D. et al., 2018), antiosteoporotic (Huang et al., 2017), cardiomyocyte-protecting (Li J. et al., 2019), and immunity-enhancing (Zeng et al., 2019) efficacy. Animal medicines have complex ingredients and it is difficult to isolate and identify effective components. Many researchers are actively engaged in the analysis and identification of ingredients of PAEs and have made some progresses. It has been reported that (Lv N. et al., 2017; Li et al., 2020) PAEs contain polysaccharides, peptides, nucleosides, polyols, steroids, terpenes, alkaloids, flavonoids, and isocoumarins, and the effective constituents of the extracts may include polysaccharides, peptides, nucleosides. However, PAEs have not been fully explored in clinical applications as their active constituents have not been adequately purified and their molecular mechanisms are not fully understood. At present, only a few representative clinical PAEs prescriptions such as “Kangfuxin solution”, “Xinmailong injection”, “Ganlong capsule” among others are used mainly for the treatment of cutaneous lesions (Li L. J. et al., 2019), chronic heart failure (Lu et al., 2018), alimentary canal diseases (Li Q. J. et al., 2018), and chronic hepatitis B (Li and Hu, 2003). PAEs stimulate healing and inhibit hepatic fibrosis progression. However, it remained uncertain whether PAEs would promote liver regeneration as effectively as skin regeneration. Here, we used a mouse liver regeneration model after 70% PH and a hepatocyte culture to determine the beneficial effects of PAEs in liver regeneration and establish their modes of action.

## Materials and Methods

### Preparation of *Periplaneta americana* Extracts (PAEs)

The original PAEs extractum was supplied by Tengchong Pharmaceutical Co. Ltd., Baoshan, Yunnan, China. The extraction protocol of PEAs followed the patent (Li and Hu, 2003) involving Ganlong capsule, a medicine for the treatment of chronic hepatitis B clinically in China. The main ingredients are sticky sugar amino acid. Briefly, dried adult *Periplaneta americana* was coarsely crushed and the powder was soaked in 95% ethanol (1:4 w/v) for 24 h and extracted by heat reflux extraction at 80°C for 8 h. After oil-water separation, the aqueous phase was filtered and evaporated under reduced pressure down to the original PAEs extractum with ≤ 15% moisture content. This condensate was stored at -20°C until subsequent use. For intragastric administration or cell culture preparation, the material was diluted in 0.9% (w/v) saline or double-distilled water, respectively.

### HPLC Analysis

Chromatographic analysis was performed on a Waters E2695 series HPLC system (Waters, MA, USA) equipped with an ultraviolet detector 2998, using an Agilent C18 SB-AQ (4.6mm×150mm, 5μm) at a column temperature of 25°C. The flow rate and injection volume were 1 ml/min and 20 µl, respectively. The methanol–water (2:98) system was employed as the mobile phase for quantitative determination of multiple standard compounds, i.e., cytosine, uracil, cytidine, uridine, inosine, and guanosine. All these standard compounds were dissolved together by 3% methanol to form a mixed standard solution (10 μg/ml). The optimized detection wavelength was 254 nm.

The inosine standard compound (C14328000) was purchased from Dr. Ehrenstorfe Co., LTD, Germany. The other standard compounds, cytosine (CDAA-281383), uracil (CDAA-28071620), cytidine (CDAA-281384), uridine (CDAA-280717), and guanosine (CDAA-280791), were purchased from ANPEL Laboratory Technologies Co., LTD, Shanghai.

### Cell Culture

The normal hepatocyte cell line L02 was obtained from the Tumor Institute of the Third Affiliated Hospital of Kunming Medical University, Kunming, Yunnan, China. The L02 cells were cultured in Gibco RPMI 1640 Medium without HEPES and supplemented with 10% fetal bovine serum (FBS) (Thermo Fisher Scientific, Waltham, MA, USA), 100 U/ml penicillin, and 100 g/ml streptomycin (Invitrogen, Carlsbad, CA, USA) in 5% CO_2_ at 37°C.

### Cell Counting Kit-8 (CCK-8) Assay

L02 cell proliferation was measured with a CCK-8 kit (Dojindo Laboratories, Kumamoto, Japan). Briefly, cells in the logarithmic growth phase were seeded in 96-well plates at a density of 5×10^3^/well. Each well contained 100 μl culture medium. After 24 h, the cells were incubated in 100 μl culture medium without FBS for 24 h and divided into the serum-starved control group (NC) and the serum starvation + 0.1, 0.5, 1, 25, or 50 mg/ml PAEs groups. After 24, 48, and 72 h, the CCK-8 kit reagents were added to the culture plates in the dark. The cells were incubated under 5% CO_2_ at 37 °C for 40 min. The absorbances were read at 450 nm in an automated plate reader (BioTek). Each experiment was repeated at least in triplicate. Based on the instructions of the CCK-8 kit, the cell proliferation ratio was calculated as follows:

(1) $$Cell\;proliferation\;ratio\;=(A_{PAEs}-A_{b})/(A_{c}-A_{b})$$

where A_PAEs_, A_c_, and A_b_ represent the average absorbances of the PAEs group, the control group, and the blank (culture medium without FBS), respectively.

### 70% Partial Hepatectomy (PH) of C57 Mice

The present study was approved by the Medical Ethics Committee of Kunming Medical University (Kunming, Yunnan Province, China). All experimental procedures were performed in accordance with protocols approved by the Institutional Animal Care and Utilization Committee.

Healthy 8–14-week male C57 mice weighing 20–24 g were obtained from the Experimental Animal Center of Kunming Medical University. All experimental mice were maintained under standard general anesthesia with intraperitoneal (IP) 3% (v/v) chloral hydrate. The dosage was 1.2 ml/100 g BW. A midline laparotomy was performed and the left lateral and left and right median lobes were ligated and resected. Here, 70% of the mouse liver was surgically removed under sterile conditions (Nevzorova et al., 2015). The mortality rate was < 10%. The main cause of death was bleeding caused by ligation line falling off after 70% PH.

### PAEs Administration

To evaluate the effect of PAEs on liver regeneration, male C57 mice were randomly divided into the nonspecific control (NC), normal saline (NS), and low- and high-dose PAEs groups (n = 10 per group).

The PAEs oral dose of adult was no more than 18 g/d in the patent (Li and Hu, 2003) involving Ganlong capsule, a medicine for the treatment of chronic hepatitis B clinically in China. According to the body surface area (BSA) normalization method, a human equivalent dose (HED) was calculated as follows (Reagan-Shaw et al., 2008):

(2) $$HED\;(mg/kg)=Animal\;dose\;(mg/kg)\times \frac{Animal\;K_{m}}{Human\;K_{m}}$$

where the K_m_ factor is 3 for a mouse and 37 for a human. The dose of mice was no more than about 3.7 g/kg/d because oral dose of 60 kg adult was no more than 18 g/d. In the present liver regeneration experiment and in reference to previous studies by others (Zhang et al., 2013; Li, 2016; Li et al., 2016; Chang et al., 2017; Tang et al., 2018), gavage administration was adopted, in which low- and high-dose PAEs groups were set as PAEs400 (400 mg/kg/d) and PAEs800 (800 mg/kg/d), respectively.

By gavage, the NS mice were administered with normal saline and the PAEs mice were administered with 400 or 800 mg/kg/d for 10 days. At 2 h after the 10th gavage, 70% PH was performed on the NS and PAEs mice. At 48 h after 70% PH, all mice were euthanized by CO_2_ inhalation. Liver tissue samples of all mice including the NC were collected immediately.

### Hepatic Regeneration Rate (HRR) Measurement

After 70% PH, the hepatic regeneration rate (HRR) of the residual liver including the right and caudate lobes was calculated as follows:

(3) $$HRR=[W_{c}-(W_{a}-W_{b})]/[W_{a}-W_{b}]\times 100$$

(4) $$W_{a}=W_{b}/70\%$$

where W_a_ is the calculated initial weight of the whole mouse liver at the start of the 70% PH, and W_b_ and W_c_ are the actual weights of the surgically excised liver tissue and the residual liver tissue at the time of death, respectively. This original weight was calculated as a fixed proportion of the liver weight according to Y. Nevzorova et al. (2015) and M. Meier et al. (2016).

### Immunohistochemical (IHC) Staining

The right lobe was split into two parts. One section was immediately frozen in liquid nitrogen for later sequencing while the other part was fixed in 10% (v/v) neutral formaldehyde for immunohistochemical (IHC) staining. The segments were fixed overnight, embedded in paraffin, and cut into 4-µm-thick sections (Leica RM2235). Hepatocyte proliferation was estimated by IHC staining for the nuclear antigen Ki-67. This antigen is preferentially expressed during all active cell cycle phases (G1, S, G2, and M) but not in G0 (Scholzen and Gerdes, 2000). Anti-Ki-67 Rabbit pAb (WL01384a) was provided by Wanleibio Co. Ltd., Shenyang, China. Immunohistochemical staining was performed using the following protocol included with the kit: primary antibody: 1:100, 4°C, overnight; secondary antibody-biotin: 1:150, 37°C, 1 h; and streptavidin-HRP: 1:200, 37°C, 30 min. The Ki-67-positive hepatocytes (N_P_) and the total hepatocytes (N_T_) were counted in random fields at ×400 magnification. The positive rate (%) of Ki-67 expression was used to enumerate the proliferating hepatocytes in the IHC staining sections and was calculated as follows:

(5) $$Positive\;rate\;(\%)=N_{P}/N_{T}\times 100$$

### RNA Sample Preparation, cDNA Library Preparation, and Illumina Sequencing

Nine samples per group were randomly selected. Three samples were randomly mixed and three mixed samples were obtained per group. Total RNA was extracted from each mixed sample with TRIzol reagent (Invitrogen, Carlsbad, CA, USA) according to the manufacturer’s instructions. Twelve DGE libraries (NC, NS, PAEs400, and PAEs800) were processed in an Illumina gene expression sample prep kit (Illumina, San Diego, CA, USA). After cDNA library quality and quantity control, Illumina sequencing was performed in an Illumina HiSeq 2000 (Mus_musculus) at the Beijing Genomics Institute (BGI), Shenzhen, China). The SRA data have been uploaded to NCBI (BioProject: PRJNA635249).

### Mapping DEGs to the Mus_Musculus Genome

Raw reads were prepared as follows to create clean reads (Cock et al., 2010). Raw reads with adapters and unknown bases (>5%) were removed. Low-quality reads with quality values < 10 for >20% of their bases were filtered out. Clean high-quality tags were mapped to the reference genome with HISAT (Kim et al., 2015) and to the reference sequences with Bowtie2 (Langmead and Salzberg, 2012). Based on the mapping results, the RSEM analysis (Li and Dewey, 2011) was executed to quantify the gene expression level and obtain a read count of each gene in each sample. The gene expression levels were calculated by the fragments per kilobase per million reads (FPKM) method (Mortazavi et al., 2008).

### Functional Analysis of Differentially Expressed Genes (DEGs)

A differential expression analysis between the treatment and control was implemented using a rigorous algorithm. The threshold *P*-value was determined by the false discovery rate (FDR) method in multiple tests (Wang et al., 2010). FDR < 0.001 and absolute value of the log2 ratio ≥ 1 were set as the detection thresholds for genes with significant differential expression. To identify significantly enriched terms, the Mus_musculus (mm10) genome was used as the background to identify GO terms enriched within the DEG dataset *via* a hypergeometric test using a corrected *P*-value (≤ 0.05) as a threshold. A Kyoto Encyclopedia of Genes and Genomes (KEGG) pathway enrichment analysis was performed to identify significantly enriched pathways within the DEG datasets. It was compared with the genome database *via* a hypergeometric test using a corrected *P*-value (≤ 0.05) as a threshold.

### Data and Statistical Analysis

The data were compiled in a spreadsheet in MS Office Excel 2010. Statistical analyses were performed by running *t*-tests on independent samples to identify significant differences. *P* < 0.05 was considered statistically significant.

## Results

### HPLC Quantitative Analysis

The amounts of six physiological small molecules, i.e., cytosine, uracil, cytidine, uridine, inosine, and guanosine, in PAEs were determined by HPLC. These nucleosides and nucleobases were deemed to be the potential active components in PAEs in the study of intestinal barrier improvement and anti-inflammation effects (Ma et al., 2018). As shown in **Figure S1A**, these six compounds in mixed standard substances were analyzed, with a satisfied degree of separation and methodological investigation being obtained.

**Figures S1B, C** provide clear evidence that the concentrations of these six compounds in PAEs sample could be determined. Accordingly, based on the external standard method, the amounts of these six compounds, i.e., cytosine, uracil, cytidine, uridine, inosine, and guanosine, in PAEs were calculated and the results were 5.91, 6.12, 7.63, 5.97, 3.26, and 1.89 mg/g, respectively. In addition, two unknown substances were found in our study.

### Hepatocyte Proliferation *In Vitro* and *In Vivo*

**Figure 1:**
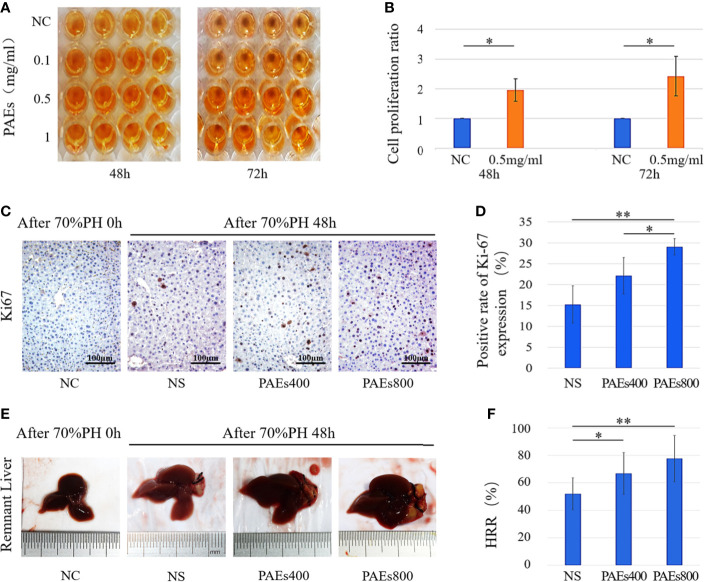
PAEs efficacy in hepatocyte proliferation both *in vitro* and *in vivo* relative to control. **(A)** Representative images of CCK-8 color reactions in 96-well plates at 48 and 72 h after treatment with different PAEs concentrations. **(B)** Differences in L02 cell proliferation ratios between PAEs treatment group (0.5 mg/ml) and NC group were significant at 48 and 72 h according to Student’s *t*-test (*P < 0.05). **(C)** Ki-67 expression levels in residual liver cell nuclei and cytoplasm at 48 h after 70% PH. **(E)** Representative images of residual liver at 48 h after 70% PH. Each group included nine mice. **(D, F)** Differences between PAEs and NS groups were significant according to Student’s *t*-test (*P < 0.05; **P < 0.01).

*In vitro*, PAEs at low dose (1 mg/ml) was found to accelerate moderate L02 cell (in Gibco RPMI 1640 Medium without HEPES and supplemented with 10% FBS) proliferation. On the other hand, virtually all cells were killed by PAEs at high doses (25 or 50mg/ml). The result was submitted as supplementary material (**Figure S2**). The CCK-8 assay showed that different PAEs concentrations (0.1, 0.5, or 1 mg/ml) accelerated L02 cell proliferation under serum starvation conditions (**Figures 1A, B**).

According to the report of Wang et al. (2011), abnormal manifestations of the test animals were not observed during the acute toxicity experiment of PAEs. The estimated LD50 was >2000 mg/kg PAEs in normal ICR mice. According to the report of Zhou (2008), LD50 was > 10 g/kg TCM powder of *Periplaneta americana* in normal female and male KM mice, and the toxicity grade of *Periplaneta Americana* was practically non-toxic. In our study, abnormal manifestations were not observed in C57 mice who were administered PAEs with 400 or 800 mg/kg. By immunohistochemical staining, Ki-67 was localized in the hepatocyte nuclei of the PAEs800 group. All postoperative groups presented with higher Ki-67 expression than the NC group (**Figure 1C**). The PAEs800 group had a significantly higher Ki-67 expression than that in the NS (*P* < 0.05) and PAEs400 (*P* < 0.01) groups 48 h after 70% PH (**Figure 1D**).

### Hepatic Regeneration Rate (HRR)

The HRR was significantly higher in the PAEs groups than the NS group 48 h after 70% PH (*P* < 0.05 or *P* < 0.01). However, the difference in HRR between the PAEs400 and PAEs800 groups (**Figures 1E, F**) was not statistically significant (*P* > 0.05).

### Quantitative Identification of Differentially Expressed Genes (DEGs) 48 h After 70% PH

**Figure 2:**
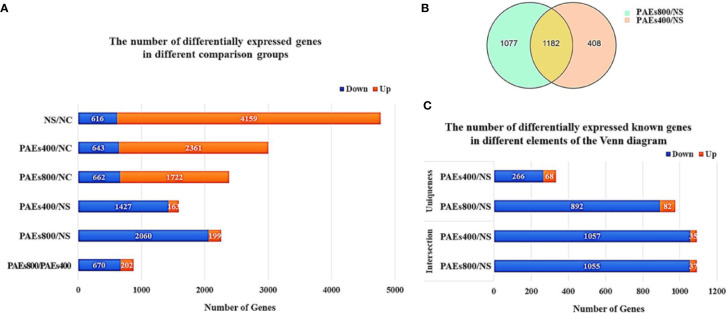
Quantitative identification of differentially expressed genes (DEGs) 48 h after 70% PH. **(A)** Number of DEGs increased or decreased by ≥ 2× (q-value < 0.001) in various comparison groups. **(B)** Venn diagram of DEGs between PAEs400/NS and PAEs800/NS. **(C)** Numbers of known DEGs in various elements of Venn diagram.

Analysis of the gene expression spectrum detected 11,986 transcripts including 9,266 coding and 2,720 noncoding transcripts. The expression levels of various genes increased or decreased by ≥ 2× (q-value < 0.001) at 48 h after 70% PH (**Figure 2A**). The PAEs groups presented with significantly fewer DEGs but more downregulated genes than the NS group.

A Venn diagram analysis identified 1,182 DEGs that were commonly expressed among the PAEs and NS groups. Moreover, 408 and 1,077 DEGs were specifically expressed in the PAEs400 and PAEs800 groups, respectively (**Figure 2B**). However, as we focused on the possible effects of the PAEs after 70% PH, we concentrated on the intersection of the 1,182 DEGs. A sequence analysis showed 1,092 reported and 90 unreported genes at the intersection. Of the former, 1,057 were downregulated and only 35 were upregulated in the PAEs400 group while 1,055 reported genes were downregulated and only 37 reported genes were upregulated in the PAEs800 group (**Figure 2C**).

### Bioinformatics Analysis of Differentially Expressed Genes (DEGs)

**Figure 3:**
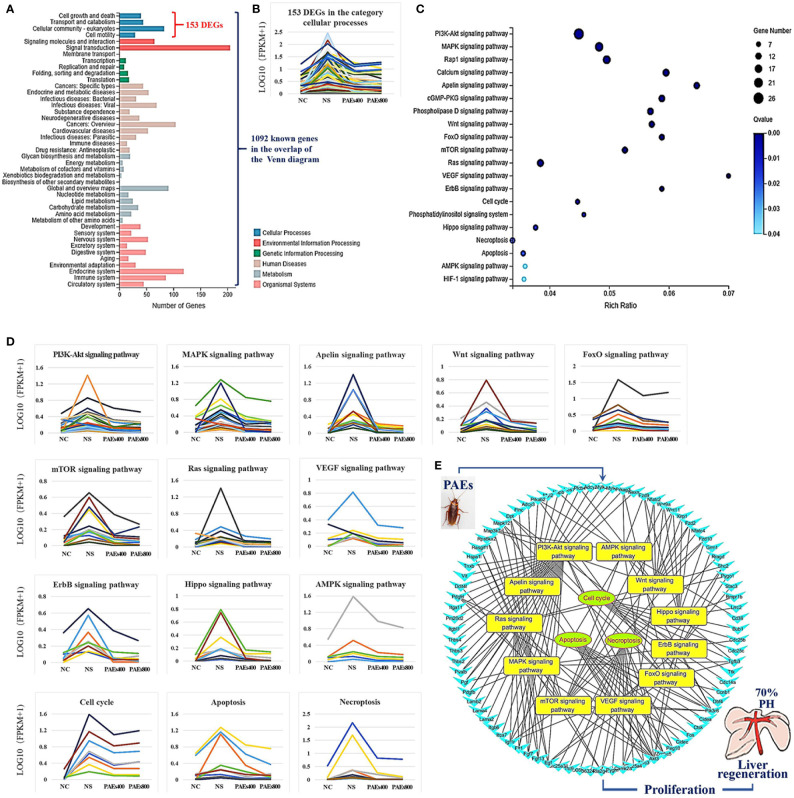
KEGG analysis of DEGs. **(A)** Annotation and classification by KEGG analysis. **(B)** Expression of 153 DEGs in the “Cellular Processes” category. **(C)** Top 20 pathways. Enrichment of 153 DEGs by KEGG pathway enrichment analysis against all identified elements. Level 2 restricted to “Cell Growth and Death” and “Signal Transduction”. Among them, 26 DEGs enriched in PI3K-AKT signaling pathway, and 21 in MAPK signaling pathway. Only seven DEGs enriched in VEGF signaling pathway, but rich ratio was the highest. This analysis result indicated an extremely complex molecular mechanism in the process by which PEAs can promote liver regeneration. **(D)** DEG expression levels in pathways. Legend shown in **Table 1**. **(E)** PAEs accelerate hepatocyte proliferation and promote liver regeneration *via* complex networks. DEG-pathway network constructed by linking 153 DEGs (blue triangles), associated signaling pathways (yellow round rectangles), and cellular processes (green ellipses).

Gene Ontology (GO) was used to annotate and enrich the DEGs and a Kyoto Encyclopedia of Genes and Genomes (KEGG) analysis was used to annotate, enrich, and analyze the signaling pathways. The KEGG pathway analysis functionally classified the 1,092 reported genes expressed in both the PAEs400 and PAEs800 treatment groups. They were categorized by the online BGI according to cellular processes, environmental information processing, genetic information processing, human diseases, metabolism, and organismal systems. **Figure 3A** shows that 153 known genes were separated into the cellular process category. Except for Pdgfrl (kinase insert domain protein receptor), the expression levels of 152 elements in the NS and PAEs groups were significantly higher than those of the NC group. However, the PAEs groups displayed intermediate expression levels. To facilitate the visualization and interpretation of gene expression from these data, we used GeneMaths LOG10(FPKM+1) to rank the DEGs according to their expression patterns and display them in concise and intelligible graphical format (**Figure 3B**). A KEGG pathway enrichment analysis identified the enriched pathways by a two-tailed Fisher’s exact test and assessed the enrichment of the 153 DEGs against all identified elements. Enrichment level 2 was restricted to “Cell Growth and Death” and “Signal Transduction” (**Figure 3C**). Pathways with corrected *P*-values ≤ 0.05 were considered statistically significant. The KEGG pathway enrichment analysis indicated that 81 DEGs participated in the cell cycle, apoptosis, and necroptosis processes and/or were involved in the signaling pathways related to cell growth or death (PI3K-Akt, MAPK, Apelin, Wnt, FoxO, mTOR, Ras, VEGF, ErbB, Hippo, and AMPK) (**Figures 3D, E**, **Table 1**). Of the 81 DEGs, 34 participated in the liver regeneration process *via* ≥ 2 signaling pathways. Akt3 was implicated in all of the aforementioned signaling pathways except for Wnt and Hippo.

**Table 1:** Genes altered by ≥ 2× between the PAEs intervention and NS groups according to the enrichment results.

	GeneSymbol	Definition	Graphic Symbol in **Figure 3D**	Number	Map ID //Map name
1	Adcy2	adenylate cyclase 2 [EC:4.6.1.1]	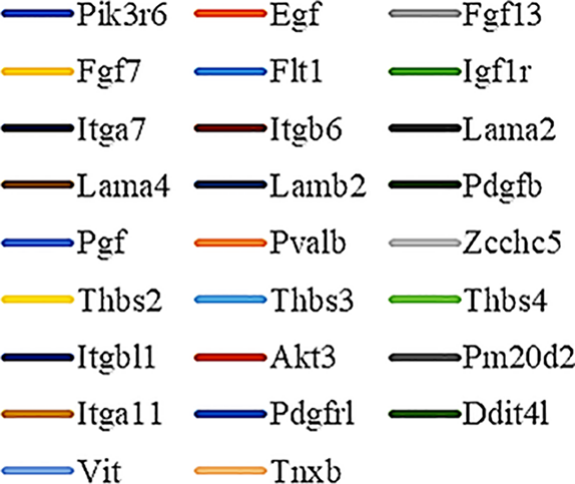	26	ko04151 //PI3K-Akt signaling pathway
2	Adcy3	adenylate cyclase 3 [EC:4.6.1.1]			
3	Akt3	RAC serine/threonine-protein kinase [EC:2.7.11.1]			
4	Bmpr1b	bone morphogenetic protein receptor type-1B [EC:2.7.11.30]			
5	Bub1	checkpoint serine/threonine-protein kinase [EC:2.7.11.1]			
6	Camk2a	calcium/calmodulin-dependent protein kinase (CaM kinase) II [EC:2.7.11.17]			
7	Ccnb1	G2/mitotic-specific cyclin-B1			
8	Cd36	CD36 antigen			
9	Cdc14a	cell division cycle 14 [EC:3.1.3.16 3.1.3.48]			
10	Cdc25b	M-phase inducer phosphatase 2 [EC:3.1.3.48]			
11	Cdc25c	M-phase inducer phosphatase 3 [EC:3.1.3.48]	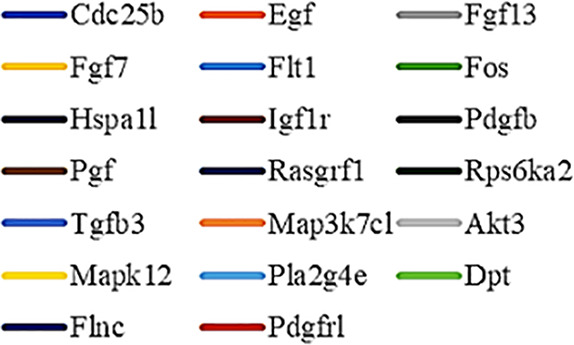	20	ko04010 //MAPK signaling pathway
12	Cidea	DNA fragmentation factor, 45 kD, alpha subunit			
13	Cidec	DNA fragmentation factor, 45 kD, alpha subunit			
14	Ctsk	cathepsin K [EC:3.4.22.38]			
15	Dbf4	activator of S phase kinase			
16	Ddit4l	DNA-damage-inducible transcript 4			
17	Dpt	Notch 1			
18	Egf	epidermal growth factor			
19	Fgf13	fibroblast growth factor	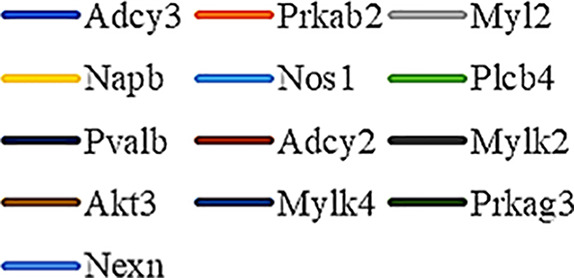	13	ko04371 //Apelin signaling pathway
20	Fgf7	fibroblast growth factor			
21	Flnc	filamin			
22	Flt1	FMS-like tyrosine kinase 1 [EC:2.7.10.1]			
23	Fos	proto-oncogene protein c-fos			
24	Fzd2	frizzled 2			
25	Fzd9	frizzled 9/10			
26	Fzd10	frizzled 9/10	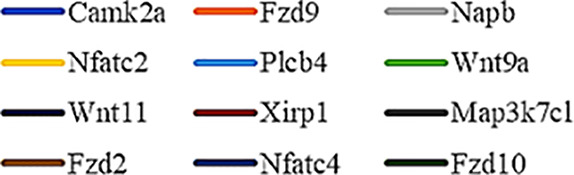	12	ko04310 //Wnt signaling pathway
27	Grm1	metabotropic glutamate receptor 1			
28	Hspa1l	heat shock 70 kDa protein 1/2/6/8			
29	Igf1r	insulin-like growth factor 1 receptor [EC:2.7.10.1]			
30	Itga7	integrin alpha 7			
31	Itga11	integrin alpha 11			
32	Itgb6	integrin beta 6			
33	Itgbl1	tenascin	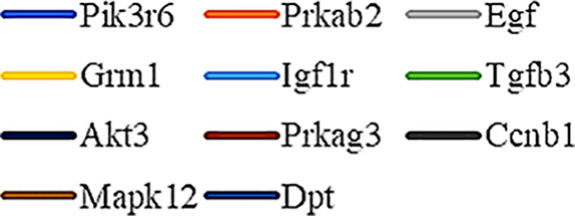	11	ko04068 //FoxO signaling pathway
34	Lama2	laminin, alpha 1/2			
35	Lama4	laminin, alpha 4			
36	Lamb2	laminin, beta 2			
37	Lrrc2	erbb2-interacting protein			
38	Map3k7cl	mitogen-activated protein kinase kinase kinase 7 [EC:2.7.11.25]			
39	Mapk12	p38 MAP kinase [EC:2.7.11.24]	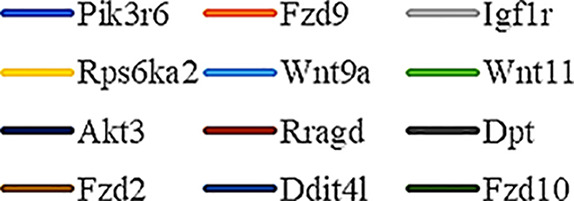	12	ko04150 //mTOR signaling pathway
40	Myl2	myosin regulatory light chain 2			
41	Mylk2	myosin-light-chain kinase [EC:2.7.11.18]			
42	Mylk4	myosin-light-chain kinase [EC:2.7.11.18]			
43	Napb	alpha-soluble NSF attachment protein			
44	Nexn	myosin-light-chain kinase [EC:2.7.11.18]			
45	Nfatc2	nuclear factor of activated T-cells, cytoplasmic 2	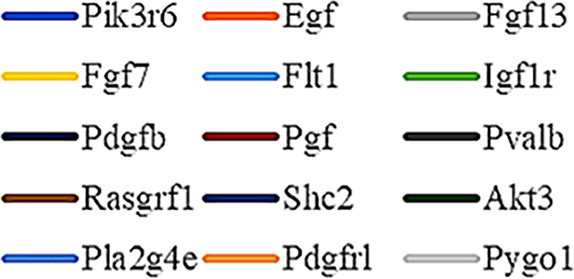	15	ko04014 //Ras signaling pathway
46	Nfatc4	nuclear factor of activated T-cells, cytoplasmic 4			
47	Nlrc3	NACHT, LRR, and PYD domains-containing protein 3			
48	Nos1	nitric-oxide synthase, brain [EC:1.14.13.39]			
49	Pdgfb	platelet-derived growth factor subunit B			
50	Pdgfrl	kinase insert domain protein receptor [EC:2.7.10.1]			
51	Peg10	poly [ADP-ribose] polymerase [EC:2.4.2.30]			
52	Pgf	placenta growth factor	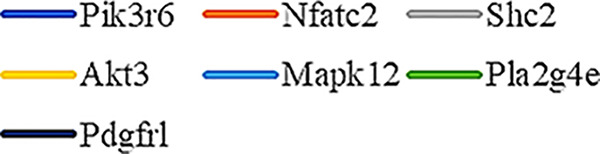	7	ko04370 //VEGF signaling pathway
53	Pik3r6	phosphoinositide-3-kinase regulatory subunit alpha/beta/delta			
54	Pla2g4e	cytosolic phospholipase A2 [EC:3.1.1.4]			
55	Plcb4	phosphatidylinositol phospholipase C, beta [EC:3.1.4.11]			
56	Pm20d2	translation initiation factor 2 subunit 3			
57	Prkab2	5’-AMP-activated protein kinase, regulatory beta subunit	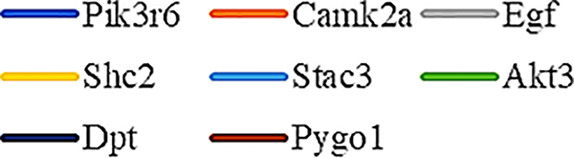	8	ko04012 //ErbB signaling pathway
58	Prkag3	5’-AMP-activated protein kinase, regulatory gamma subunit			
59	Pvalb	atrophin-1 interacting protein 1			
60	Pygm	glycogen phosphorylase [EC:2.4.1.1]			
61	Pygo1	SHC-transforming protein 1			
62	Rasgrf1	Ras-specific guanine nucleotide- releasing factor 1	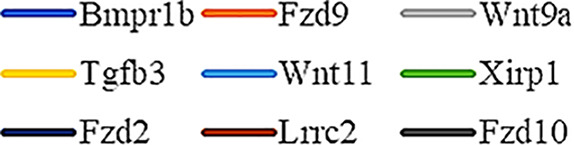	9	ko04390 //Hippo signaling pathway
63	Rps6ka2	ribosomal protein S6 kinase alpha-1/2/3/6 [EC:2.7.11.1]			
64	Rragd	Ras-related GTP-binding protein C/D			
65	Shc2	SHC-transforming protein 2			
66	Slc25a31	solute carrier family 25 (mitochondrial adenine nucleotide translocator), member 4/5/6/31			
67	Slc25a4	solute carrier family 25 (mitochondrial adenine nucleotide translocator), member 4/5/6/31	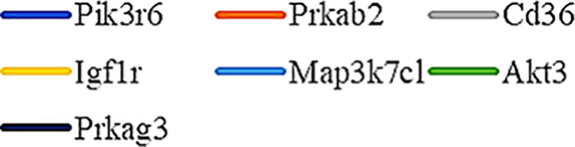	7	ko04152 //AMPK signaling pathway
68	Stac3	signal transducing adaptor molecule			
69	Tgfb3	transforming growth factor beta-3			
70	Thbs2	thrombospondin 2/3/4/5	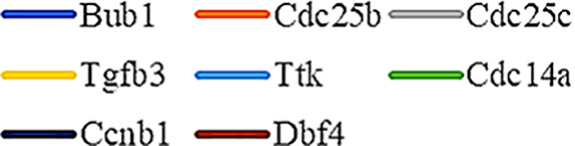	8	ko04110 //Cell cycle
71	Thbs3	thrombospondin 2/3/4/5			
72	Thbs4	thrombospondin 2/3/4/5			
73	Tnxb	tenascin			
74	Ttc9	peptidyl-prolyl isomerase D [EC:5.2.1.8]			
75	Ttk	serine/threonine-protein kinase TTK/MPS1 [EC:2.7.12.1]	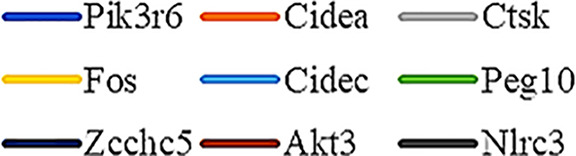	9	ko04210 //Apoptosis
76	Vit	collagen, type VI, alpha			
77	Wnt9a	wingless-type MMTV integration site family, member 9			
78	Wnt11	wingless-type MMTV integration site family, member 11			
79	Xirp1	methyl CpG binding protein 2	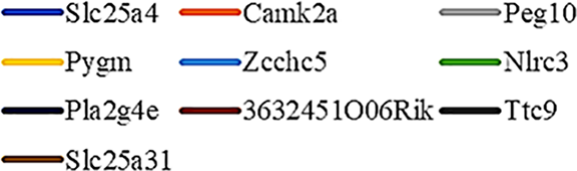	10	ko04217 //Necroptosis
80	Zcchc5	poly [ADP-ribose] polymerase [EC:2.4.2.30]			
81	3632451 O06Rik	high mobility group protein B1			

## Discussion

*Periplaneta americana* (PA) is a common source of animal medicine that has a long history of use in TCM for the treatment of wounds and burns (Alves and Alves, 2011; Zhu et al., 2018). Over the past two decades, PAEs have been tested for the promotion of gastric and duodenal ulcer healing (Lu et al., 2019), treatment of hepatic fibrosis (Li D. et al., 2018), inhibition of tumor growth (Zhao et al., 2017), stimulation of skin wound healing (Yang et al., 2015; Song et al., 2017), and induction of skin fibroblast migration (Li L. J. et al., 2019). The active constituents in PAEs with wound healing efficacy have been screened (Li Q. J. et al., 2018). The abovementioned studies, however, did not provide evidence for the healing potential of PAEs. To the best of our knowledge, the present study is the first to report on the liver regeneration efficacy of PAEs involving the synergy of multiple signaling pathways.

The present results have shown that PAEs promote hepatocyte proliferation both *in vitro* and *in vivo* and accelerate mouse liver regeneration after 70% partial hepatectomy. That PAEs can accelerate liver regeneration by affecting hepatocyte proliferation is evident by the increase in incidence of Ki-67 immunohistochemical stained hepatocyte nuclei. The DEGs bioinformatics analysis revealed that PAEs influence the expression levels of different genes after PH. On closer analysis, some of the DEGs participated in cellular processes and signaling pathways associated with cell growth or death. The cellular processes included cell cycle, apoptosis, and necroptosis. The signaling pathways included PI3K-Akt, MAPK, Apelin, Wnt, FoxO, mTOR, Ras, VEGF, ErbB, Hippo, and AMPK (**Figure 3E**).

The murine 70% PH model is not associated with tissue injury or inflammation because the liver lobes are removed intact. Of note, the expression levels of the DEGs related to cell death were lower in the PAEs groups than the NS group. It stands to reason therefore that PAEs may protect hepatocytes by inhibiting cell death after liver injury. They may promote post-PH liver regeneration by inducing the proliferation of existing mature hepatocytes without activating the progenitor cells (Mao et al., 2014). Though the PAEs accelerated liver regeneration, the expression levels of the DEGs related to cell growth were lower in the PAEs group than the NS group. It is suggested that they may be concerned more with the process of liver regeneration after 70% PH.

After 70% PH, most of the hepatocytes in the residual lobes undergo one or two proliferative processes (Michalopoulos and DeFrances, 2005). In rats, the first peak in hepatocyte DNA replication occurs after ~24 h. A second smaller peak is observed between 36–48 h and there is a maximum rate of cell division at 36 h after PH. In contrast, there is species variation in peak DNA replication following PH; in mice, the peak was 12‑16 h later compared to rats (Mao et al., 2014). Nevzorova et al. (2015) suggested that for studies on the progression phase, the mice should be sacrificed at 36 (S-phase onset), 40, 48 (peak of DNA synthesis), and 60 h (termination of cell cycle activity) after PH. Here, we collected the liver samples at 48 h after liver resection. A peak in DNA replication and significant upregulation of the DEGs related to cell growth occurred in NS at this time point. There were comparatively higher HRR and relatively lower expression levels of the DEGs associated with cell cycle and proliferation in the PAEs groups. Hence, PAEs accelerate the initial hepatocyte proliferation process and hasten the arrival of the cells to the second proliferative event. PAEs improved L02 cell proliferation *in vitro;* thus, they possess liver regeneration potential.

Previous studies had explored the effects of single factors and pathways on liver regeneration. However, more recent reports demonstrated that numerous signaling pathways are involved in this process and their interactions are complex. Gupta et al. (2019) found that ALR (augmenter of liver regeneration, a protein that was identified to specifically support liver regeneration) induced miRNA-26a expression, upregulated the p-Akt/cyclin D1 pathway, and promoted hepatic cell proliferation. Lai et al. (2015) found that EGR-1 (early growth response 1 gene)-induced GGPPS plays an important role in post-PH liver regeneration *via* RAS/MAPK signaling. Ye et al. (2018) reported that Diwu Yanggan capsules can improve the liver regeneration microenvironment by regulating the Ras/Raf/Mek/Erk signaling pathway and regeneration-related factors. It has been reported in knockout mice or use of inhibitors that single-pathway disruption does not block but causes delay in regeneration. This suggests that a complex pathway network is vital for optimal liver regeneration and the generation of adequate hepatic mass (Michalopoulos, 2007; Mao et al., 2014). In an IL-6^-/-^ mouse model, liver regeneration was delayed (Cressman et al., 1996). Gene expression and hepatocyte proliferation can be corrected with a preoperative IL-6 injection. After PH, Tnfr1^-/-^ mice presented with multiple liver regeneration defects whereas Tnf^-/-^ mice underwent normal liver regeneration. Thus, other ligands may bind to TNFr1 (Yamada et al., 1997; Hayashi et al., 2005). In practice, pathophysiological processes are seldom induced by isolated changes in single molecular effectors. Indeed, physiological and pathological modulations are nearly always the result of the complex interactions of multiple signaling pathways.

**Figure 4:**
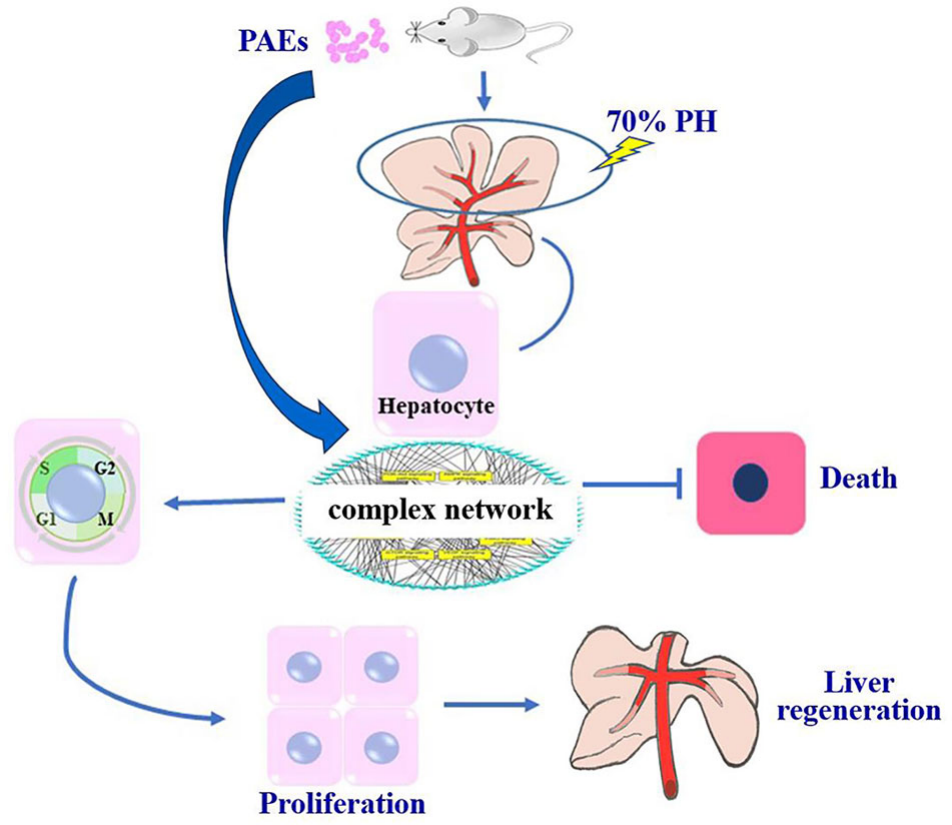
PAEs accelerate liver regeneration *via* a complex network after 70% PH in mice.

PAEs are prepared from *Periplaneta americana* and contain various biologically active ingredients including polysaccharides, peptides, nucleosides, polyols, steroids, terpenes, alkaloids, flavonoids, and isocoumarins. The effective constituents may be attributed to polysaccharides, peptides, nucleosides, among others (Lv N. et al., 2017; Li et al., 2020). Therefore, PAEs may have multiple molecular targets and biochemical properties. In our study, the main ingredients of PAEs are sticky sugar amino acid, based on the patent (Li and Hu, 2003) involving Ganlong capsule; however, they are still not considered a natural pure compound drug. The efficacy of PAEs at accelerating liver regeneration after 70% PH was mediated by the synergistic effect of numerous targets and pathways associated with cell growth and death. These pathways can form complex interconnecting networks. In fact, certain DEGs participated in ≥ 2 pathways (**Figure 4**). This discovery corroborates the synergy of action theory of multicomponent therapeutics TCM postulated by Lv C. et al. (2017). In the latter, the gene expression profiles of four active constituents in Danshen were analyzed. In this connection, molecular analysis of the expressions of the key signaling members by qPCR and western-blotting using L02 cells culture *in vitro* along with animal experimentation is clearly desirable. This would help unravel the major pathways and explore the mechanism that guide the enhancement of liver regeneration by the active ingredients of PAEs.

Though the liver can recover following hepatectomy, liver failure will nonetheless occur if the tissue loss is very extensive. This response is known as the Small-for-Size-Syndrome (SFSS). Tschuor et al. (2016) reported that deficient liver regeneration is the principal cause of SFSS. Successful regeneration may lead to recovery from acute liver failure. However, no regenerative strategies for existing acute liver failure have been established (Kojima et al., 2020). It is unequivocal from the present results that PAEs administered prior to surgery could enhance the postoperative liver regeneration capacity. Hence, PAEs might have clinical potential for the treatment of post-hepatectomy liver failure.

The previous study in mice showed that the toxicity grade of *Periplaneta Americana* was practically non-toxic (Zhou, 2008). In our liver regeneration experiment, the mortality rate was <10%. The main cause of death was bleeding caused by ligation line falling off after 70% PH. Abnormal manifestations were not observed in mice who were administered PAEs. Based on the BSA normalization method (Reagan-Shaw et al., 2008), human equivalent dose of PAEs (about 3.9 g/d), which was converted from the high dose (800 mg/kg) of mice, was less than 1/4 of the safe dose (18 g/d) (Li and Hu, 2003). In the experiment, the doses of PAEs used were safe and effective. This indicates that PAEs are safe and indeed have the potential for clinical application.

## Conclusions

To the best of our knowledge, the present study is the first to demonstrate that preoperative PAEs gavage increased postoperative liver regeneration capacity in mice. In the present liver regeneration experiment, the doses of PAEs used were safe and effective. This indicates that PAEs are safe and indeed have the potential for clinical application. More importantly, we show here that PAEs improved liver regeneration *via* a complex network of targets and signaling pathways. Though the present therapeutic approach using PAEs has practical merit, significance, and potential, the molecular targets, active constituents, and modes of action have not yet been elucidated. This will certainly be the scope of our future study.

## Data Availability Statement

The sequencing data has been deposited into the Sequence Read Archive (accession: PRJNA635249).

## Ethics Statement

The animal study was reviewed and approved by Medical Ethics Committee of Kunming Medical University.

## Author Contributions

XZ, XW, and KW designed the project and helped analyze the data and finalize the manuscript. YZ, MZ, and DZ performed most of the experiments, participated in discussions and data analysis, and prepared the first draft of the manuscript. YR and LS helped analyze the main data and revise the manuscript. ZM, JZ, and CX conducted certain experiments and maintained the experimental mice. ZY, ZQ, and RX performed tissue paraffin embedding, sectioning, and immunohistochemical (IHC) staining. SL, QK, HZ, SZ, and LL assisted with animal surgery and tissue sample excision.

## Funding

The present study was supported in part by the National Natural Sciences Foundation of China (Grant No. 81760430) and the Yunnan Provincial Department of Education (Grants No. 2020J0137 and 2018Y040).

## Conflict of Interest

The authors declare that the research was conducted in the absence of any commercial or financial relationships that could be construed as a potential conflict of interest.

## Supplementary Material

Figure S1 HPLC quantitative analysis. **(A)** HPLC chromatograms of mixed standard substances. **(B)** HPLC chromatograms of PAEs samples. **(C)** The merge of A and B. Peaks 1∼6 are derived from cytosine, uracil, cytidine, uridine, inosine, and guanosine, respectively. Two unknown substances were found in our study.

Figure S2 The toxicity profile of the PAEs *in vitro*. **(A)** Representative images of CCK-8 color reactions in 96-well plates at 24 and 48 h after treatment with different PAEs concentrations (in Gibco RPMI 1640 Medium without HEPES and supplemented with 10% FBS). **(B)** PAEs at low dose (1 mg/ml) was found to accelerate moderate L02 cell proliferation. On the other hand, virtually all cells were killed by PAEs at high doses (25 or 50 mg/ml).
