# Supplementary figures and images for: Periplaneta americana Extracts Accelerate Liver Regeneration via a Complex Network of Pathways
Source: Front Pharmacol. 2020 Jul 31;11:1174. doi: 10.3389/fphar.2020.01174 (PMC7413023; doi:10.3389/fphar.2020.01174)

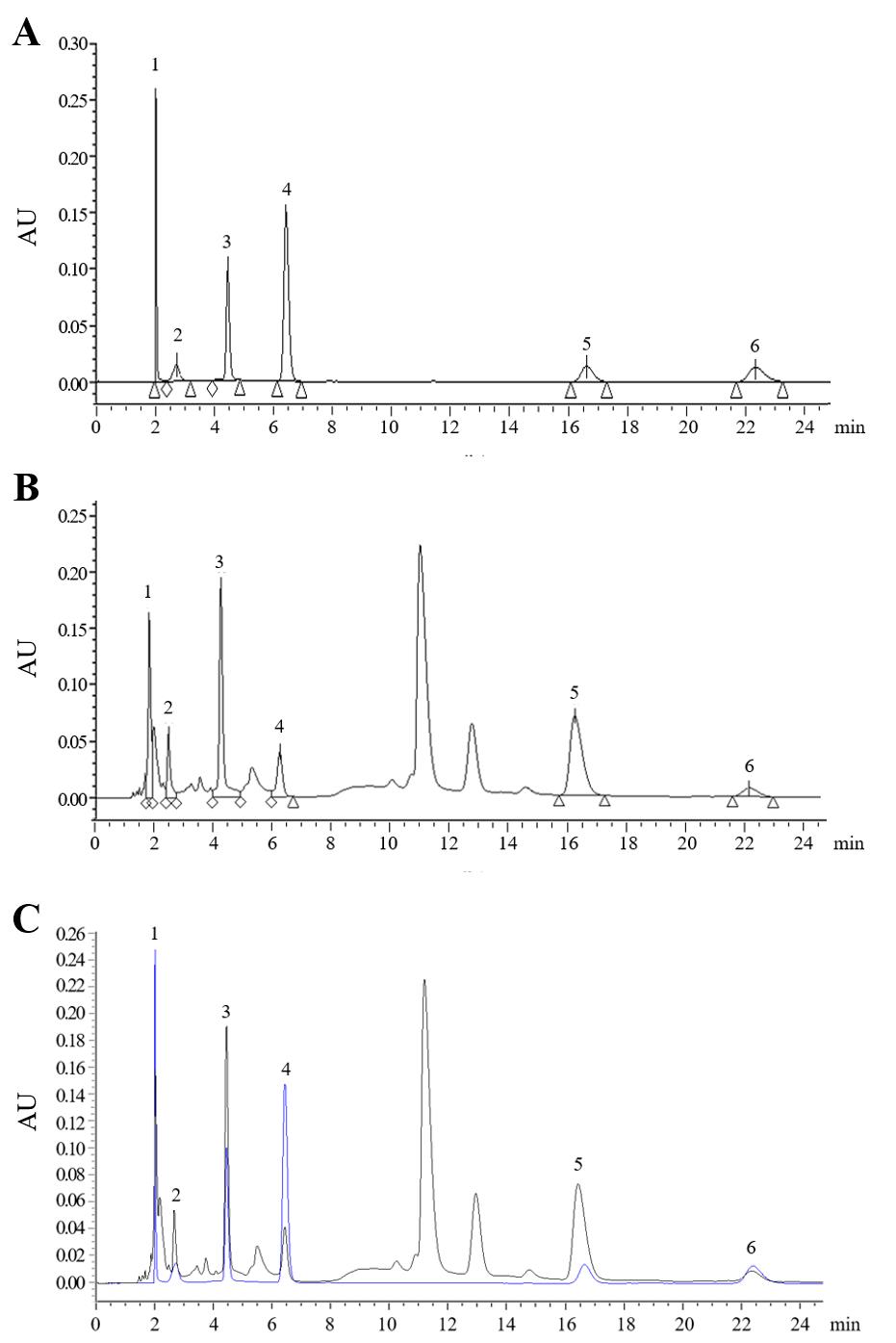

Supplement: Figure S1 — HPLC quantitative analysis. (A) HPLC chromatograms of mixed standard substances. (B) HPLC chromatograms of PAEs samples. (C) The merge of A and B. Peaks 1∼6 are derived from cytosine, uracil, cytidine, uridine, inosine, and guanosine, respectively. Two unknown substances were found in our study. [file Image_1.jpeg]

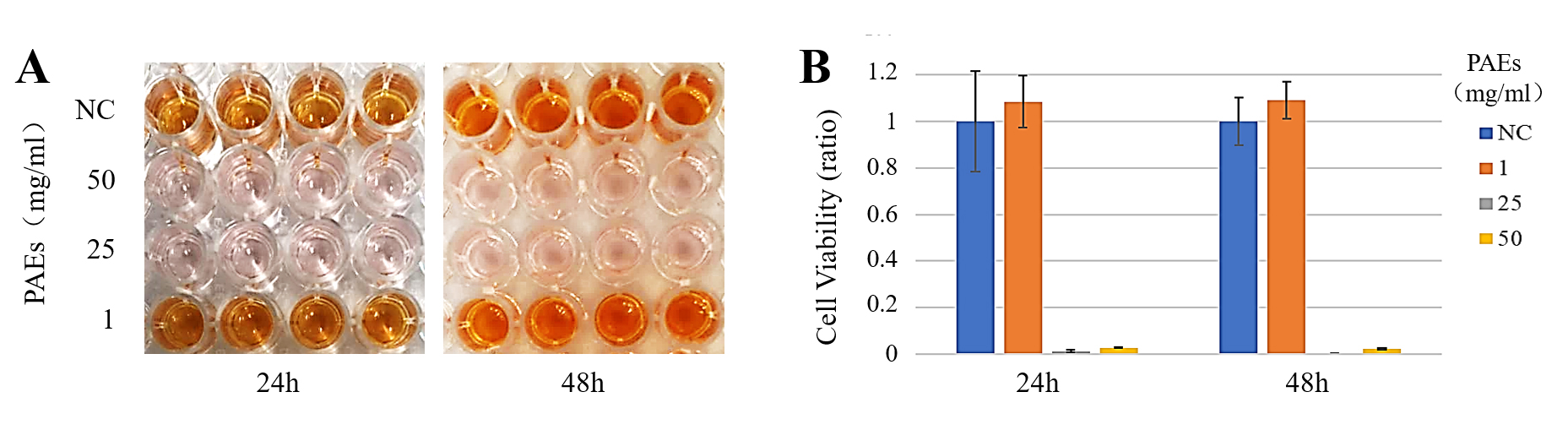

Supplement: Figure S2 — The toxicity profile of the PAEs in vitro. (A) Representative images of CCK-8 color reactions in 96-well plates at 24 and 48 h after treatment with different PAEs concentrations (in Gibco RPMI 1640 Medium without HEPES and supplemented with 10% FBS). (B) PAEs at low dose (1 mg/ml) was found to accelerate moderate L02 cell proliferation. On the other hand, virtually all cells were killed by PAEs at high doses (25 or 50 mg/ml). [file Image_2.jpeg]
